# Microscopic Characterization of Radiation Resistance of Epoxy Resin Enhanced with Graphene Quantum Dots and Molecular Simulation

**DOI:** 10.3390/ma18184303

**Published:** 2025-09-14

**Authors:** Liang Zou, Xinya Luo, Zhiyun Han, Zhen Li, Xiaofeng Ding, Kejie Huang, Hanwen Ren

**Affiliations:** 1 School of Electrical Engineering, Shandong University, Jinan 250061, China; zouliang@sdu.edu.cn (L.Z.); 202314645@mail.sdu.edu.cn (X.L.); 202420753@mail.sdu.edu.cn (Z.L.); 2 School of Automation Science and Electrical Engineering, Beihang University, Beijing 100191, China; dingxiaofeng@buaa.edu.cn; 3 State Key Laboratory of HVDC, Electric Power Research Institute, CSG, Guangzhou 510663, China; huangkj@csg.cn; 4 State Key Laboratory of Alternate Electrical Power System with Renewable Energy Sources, North China Electric Power University, Beijing 102206, China; rhwncepu@ncepu.edu.cn

**Keywords:** epoxy resin, graphene quantum dot, molecular dynamic, radiation-resistant modification design

## Abstract

With the development of the new energy industry in high-altitude regions, epoxy resin insulating materials in electrical equipment face severe challenges from prolonged exposure to strong radiation environments. Strong ultraviolet irradiation induces the generation of free radicals such as alkyl (CH_2_), alkoxy (CH_2_O), and peroxyl (CH_2_OO), which continuously attack the cross-linking structure of epoxy resin, leading to its degradation. This study employs molecular dynamics simulations to evaluate the enhancing effect of graphene quantum dots (GQDs) on the radiation resistance of epoxy resin (EP), proposing cross-linking structural integrity as an evaluation criterion. It compares and analyses pure EP (EP/neat), hydrogen-terminated GQDs (EP/GQD_C_54_H_18_), and carboxyl-terminated GQDs (EP/GQD_COOH) under three types of free radicals. The results indicate that the unique sp^2^ hybrid structure and hydrogen-donating ability of GQDs can effectively inhibit the activity of free radicals, and improve the integrity of the cross-linked structure by 8% to 16% compared to EP/neat. While both types of GQDs demonstrate comparable behavior in response to alkyl free radicals, EP/GQD_COOH exhibits superior performance under the influence of oxygen-containing free radicals. This enhanced performance can be attributed to its augmented hydrogen-donating capacity and an increased number of active sites. This study investigates the extent to which GQDs with different structures enhance the radiation resistance of epoxy resins, providing an important theoretical basis for the modification of epoxy resins for applications in high-radiation environments.

## 1. Introduction

As a general term for high-molecular-weight polymers containing epoxy groups, epoxy resins are widely used in electrical and electronic equipment such as reactors and wind turbine blades due to their excellent mechanical properties, high dielectric properties, strong corrosion resistance, and ease of processing [1,2,3,4]. The accelerating development of the new energy power industry in high-altitude regions is concomitant with the deepening of global new energy trends [5]. Epoxy resin, a critical insulating material for a variety of electrical equipment, must demonstrate stability in high-altitude regions to ensure reliable performance and safety. However, high-altitude areas are generally characterized by strong ultraviolet radiation. Prolonged exposure to ultraviolet radiation induces the generation of free radicals in epoxy resins, which trigger a series of chemical reactions that destroy the cross-linking structure of the epoxy resin, leading to material degradation and deterioration of insulation performance, thereby seriously jeopardizing the normal operation of power equipment [6]. Consequently, enhancing the radiation resistance of epoxy resin is imperative for ensuring the reliable operation of power equipment in high-altitude regions.

To enhance the radiation resistance of polymers such as epoxy resins, researchers have proposed the incorporation of nanofillers into the polymer matrix to augment its radiation resistance. Emayaruba G. Barathi Dassan et al. found that carbon nanotube-doped materials can effectively resist radiation exposure [7]. Vimal K Tiwari et al. found that the dispersion of layered silicates in polymer matrices forms intercalated nanostructures, which can effectively mitigate the degradation behavior of thermoplastic materials under irradiation [8]. Xia Wei et al. modified the graphene surface with silane coupling agents to prepare graphene/epoxy composite coatings. Electron Spin Resonance (ESR) results showed that graphene dispersed in the epoxy matrix could effectively adsorb some free radicals [9]. Although the above materials can improve the radiation resistance of polymers to a certain extent, their use is limited due to the need for strong acids or strong alkalis in the actual preparation process and poor surface compatibility of the polymers [10].

Graphene quantum dots (GQDs) are zero-dimensional materials formed when two-dimensional graphene is reduced in size to a few nanometers. The lateral diameter of these particles is typically less than 10 nm. In addition to possessing the exceptional properties of graphene, these materials exhibit a high specific surface area, a substantial π-conjugated system, and modifiable edge groups [11]. These characteristics result in stronger quantum confinement effects and edge effects, making GQDs widely applicable in fields such as solar cells and electronic devices [12]. The natural sp^2^ hybridization, hydrogen-donating surface functional groups, and structural defects of GQDs confer upon them the capacity for free radical scavenging. Yingmin Wang prepared GQDs with varying surface functional groups through multiple methods and examined the correlation between these functional groups and their antioxidant activity [13]. Hou JinXia et al. prepared GQD/EP composite materials to study the radiation resistance of GQDs as free radical scavengers. The experimental results showed that the introduction of GQDs reduced the number of free radicals after irradiation [10]. As demonstrated by Chen et al., alterations in the microstructure of epoxy resins have been shown to exert a significant influence on their macroscopic properties [14]. Liu Shengkai et al. found that the incorporation of GQDs has been shown to enhance the macroscopic property of radiation damage resistance. Regarding mechanical properties, GQDs/EP demonstrated a retention of approximately 67% of its mechanical strength following irradiation, while pure EP exhibited a retention of only 51% [15]. Consequently, alterations in the micro-crosslinked structure of epoxy resin are closely correlated with its macro-performance. The investigation of the protective effect of GQDs on the microstructure of epoxy resin under conditions of radical damage is of significant importance. However, the extant studies have predominantly focused on the limitation of GQDs on the generation of free radicals, lacking a quantitative assessment of the inhibitory performance of GQDs on the subsequent destruction of epoxy resin structures by free radicals. Moreover, a comparative assessment of the radiation resistance of epoxy resins modified by doping with GQDs of different structures has not been conducted.

A substantial body of research has been dedicated to investigating the radiation resistance of EP/GQDs composite materials through experimental methodologies. However, the preparation processes for graphene quantum dots with different structures are complex and costly, making them inefficient for researching and designing modification schemes [16]. Furthermore, the alterations in the microscopic cross-linking structure of EP/GQDs following irradiation are challenging to directly observe and evaluate through conventional experimental methods. To date, there are no direct quantitative assessment criteria available for evaluating the enhancement of epoxy resin’s radiation resistance by graphene quantum dots modified with different functional groups. Molecular dynamics (MD), a method that has been demonstrated to characterize the formation and transformation of chemical bonds between material structures, enables the observation of structural changes in EP/GQDs composite materials at the microscopic level. Accordingly, the present study employs molecular dynamics to simulate the damaged behavior of free radicals generated by ultraviolet irradiation on EP/GQDs composite materials at the microscopic level. The present study utilizes a comparative analysis of the extent of cross-linking structural damage in epoxy resin and graphene quantum dot/epoxy resin composite materials following free radical damage to evaluate the extent to which graphene quantum dots enhance the radiation resistance of epoxy resin. This analysis provides a novel theoretical foundation for the modification design of epoxy resin in terms of radiation resistance.

## 2. Materials and Simulation

### 2.1. Construction of Materials Model

**Figure 1:**
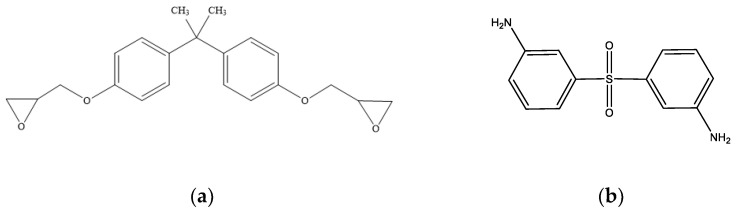
(**a**) Bisphenol A diglycidyl ether (DGEBA); (**b**) 3,3′-diaminodiphenylsulphone (33DDS).

Epoxy resins, a type of thermoplastic polymer, are typically formed by cross-linking epoxy resin monomers with curing agents. In this paper, bisphenol A diglycidyl ether (DGEBA) is selected as the epoxy resin monomer, and 3,3′-diaminodiphenylsulphone (33DDS) acts as the curing agent. Their molecular formulas are shown in Figure 1.

**Figure 2:**
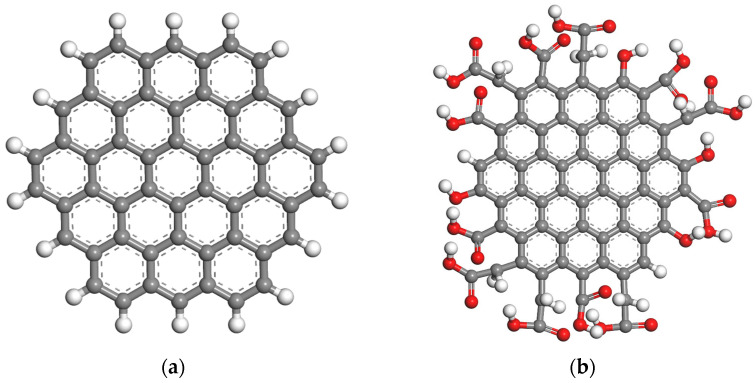
Two types of GQD with different terminal groups: (**a**) GQD_C_54_H_18_ with H groups at the end; (**b**) GQD_COOH with oxygen-containing groups at the ends.

In the actual preparation of epoxy resin, due to the extremely small amount of catalyst added, to simplify the simulation process, only the cross-linking reaction between the epoxy resin monomer and the curing agent is considered. The amino groups contained within the curing agent have the capacity to react with two epoxy groups, thereby ultimately forming a cross-linked polymer network of epoxy resin. Consequently, the reaction ratio of epoxy resin to curing agent is 2:1. According to the method proposed by K.M.M. Méndez-Martínez et al. for constructing molecular models of graphene quantum dots [17], starting from the simplest aromatic structure C_6_H_6_, i.e., a benzene molecule, aromatic rings are added sequentially to ultimately form a graphene quantum dot structure with C_54_H_18_, with H at both ends, as shown in Figure 2a. A prevalent “top-down” hydrothermal preparation technique employed in actual production yields GQDs with oxygen functional groups. The methodology employed by Yongqiang Dong et al. enables the construction of GQDs with carboxyl functional groups, as illustrated in Figure 2b [18].

**Figure 3:**
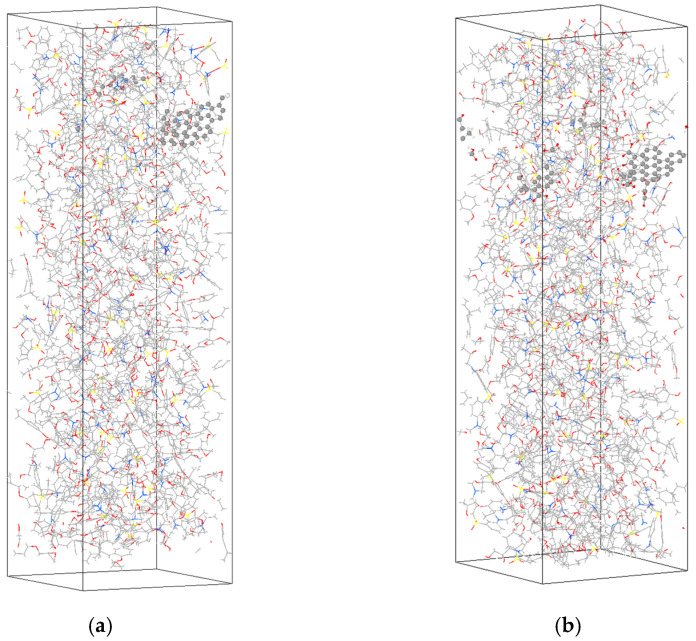
The two EP/GQD doping models with grey representing C, white representing H, red representing O, yellow representing S, and blue representing N.: (**a**) EP/GQDs_ C_54_H_18_; (**b**) EP/GQD_COOH.

To ensure the simulated reaction possesses sufficient space for reaction and conforms to actual density, this paper selected 120 DEGBA and 60 33DDS for cross-linking modeling. The two types of GQDs are, respectively, doped into epoxy resin. The mass percentages of GQD_C_54_H_18_ and GQD_COOH are 1.18% and 2.35%, respectively. The difference in model mass percentages primarily stems from the distinct functional group modifications at the ends of the graphene quantum dots. Based on the cross-linking epoxy resin modeling method proposed by Wu C. [19], the cross-linking reaction sites are automatically connected using the cross-linking script and modeling software to achieve complex epoxy resin cross-linking modeling, thereby establishing composite material models of different structures for EP/GQDs. Two models of epoxy resin composite materials doped with graphene quantum dots are shown in Figure 3. Subsequently, the Reaxff molecular simulation force field [20] is employed to perform kinetic relaxation on four epoxy resin models using the LAMMPS molecular simulation software (Lammps-17Apr2024) [21,22] at a temperature of 300 K. Ultimately, a stable equilibrium system is obtained to serve as the system model for subsequent computational simulations.

### 2.2. Simulation of Settings

The predominant form of damage inflicted by ultraviolet irradiation on epoxy resins is the generation of free radicals, which engage in irreversible chemical reactions with the epoxy resin, consequently disrupting its cross-linking structure. Preliminary research suggests that the types of free radicals induced in epoxy resins under irradiation are primarily alkyl, alkoxy, and peroxyl radicals [23,24,25]. In this study, CH_2_, CH_2_O, and CH_2_OO are selected as representatives of the three different free radical types. Due to differences in free radical concentrations produced under different irradiation conditions, based on the electron spin resonance spectroscopy results of Zhenyan Ji et al., it can be seen that for every 1000 kGy of irradiation absorbed by epoxy resin, 3.2 × 10^20^ spins/g of unpaired electrons are produced [25]. To illustrate this phenomenon, consider the annual irradiance flux of 5400 MJ/m^2^ in China’s Qinghai–Tibet Plateau region [26]. According to the epoxy resin model developed in this study, the absorption of this annual irradiance by the model results in the generation of approximately 62 unpaired electrons. Given that the three types of free radicals each contain approximately 2–3 unpaired electrons, the total number of free radicals produced is approximately 33.6. For the sake of convenience in calculation and simulation, this study ultimately selected 35 free radicals as the benchmark parameter. When free radicals are first generated, they absorb energy. The dissociation energy of covalent bonds in epoxy resins is approximately 300–400 kJ/mol, and the resulting free radicals generally have an energy of 3–4 eV [27].

**Figure 4:**
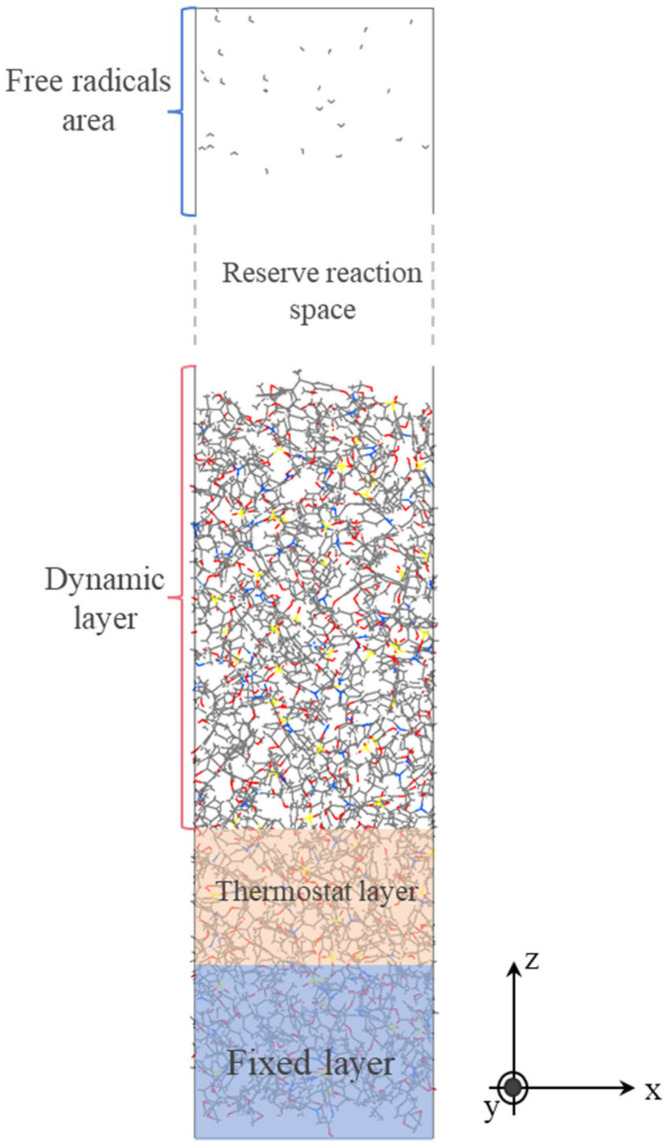
The model of epoxy resin and free radicals.

This study aims to investigate the inhibitory effect of graphene quantum dots on free radical erosion of epoxy resin. Given the highly reactive nature of free radicals and the need for stable simulation conditions, this study constructed three layers with different simulation conditions in a simulated actual setting, divided into: a fixed layer, a constant temperature layer, and a moving layer. The atoms within the fixed layer are devoid of velocity, thereby ensuring that the substrate remains inviolate and atoms are not expended. The constant temperature layer utilizes Langevin temperature control to ensure stable temperature and effective heat dissipation. The moving layer functions as the reaction layer, and the NVE system is employed to observe structural changes in epoxy resin under free radical damage. The periodic boundary constraint in the *Z*-axis direction was removed, and a vacuum layer of approximately 70 Å is constructed to store the free radicals required for the simulation. The final model incorporating the free radical region and detailed partition settings is shown in Figure 4. The simulation is divided into two stages. In the first stage, 30 free radicals are temporarily stored in the vacuum layer region along the *Z*-axis. Then, every 2000 steps (0.1 fs/step), the free radicals are injected into the epoxy resin model. In the second stage, a total of 150 ps of molecular dynamics simulation is conducted after all free radicals have entered the epoxy resin, outputting atomic trajectory files and bonds.reaxff files.

## 3. Results

### 3.1. Microscopic Assessment Indicators for Cross-Linking Structure Damage

**Figure 5:**
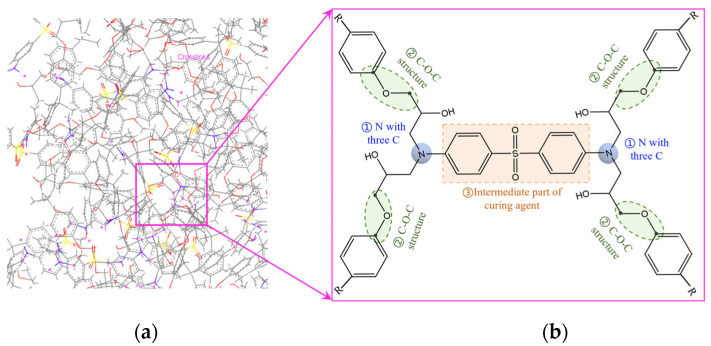
(**a**) The enlarged image of cross-linking model; (**b**) the unit cross-linking structure.

When epoxy resin is exposed to radiation, its macroscopic properties, such as strength, modulus, dielectric properties and thermal stability, change. This is fundamentally due to free radicals disrupting the resin’s cross-linking structure, thereby leading to performance degradation [14,28]. Therefore, it is of paramount importance to investigate the protective effect of GQDs on cross-linking structures. In Section 2.1 of this study, a cross-linking reaction between the epoxy resin and the curing agent was performed using a cross-linking script to form a three-dimensional network structure. The results of the cross-linking model are presented in Figure 5. Figure 5a shows a magnified view of a region within the model after cross-linking completion. In this figure, C atoms are depicted in gray, S in yellow, O in red, and N in blue. The cross-linking sites are indicated by purple dots. The final three-dimensional network structure of the epoxy resin consists of numerous interconnected basic cross-linking units. A representative basic cross-linking unit is illustrated in Figure 5b. When fully reacted, the amine groups in the curing agent should react with two epoxy groups. Therefore, in a completely basic cross-linking unit, a N atom should be bonded to three C atoms, as indicated by (a) in Figure 5. However, incomplete cross-linking reactions can occur in practice. In such cases, an amine group may react with only one epoxy group, resulting in a nitrogen atom bonded to only two carbon atoms. The C-O-C linkage connecting the benzene ring and the cross-linking sites serves as an important backbone segment of the cross-linking structure, shown by (b) in Figure 5. The sulfur-containing part in the curing agent acts as a bridge, connecting multiple epoxy resin units bonded to nitrogen atoms, thereby constructing the cross-linking network structure. Thus, the formation of the epoxy resin’s cross-linked network primarily relies on the backbone C-O-C linkages and the N-C bonds formed by the reaction between nitrogen atoms and epoxy groups. These two types of bonds are crucial for constructing the epoxy resin’s cross-linking network. A reduction in the number of C-O-C bonds and N-C bonds signifies damage to the cross-linking structure.

In this study, a 150 ps MD simulation was performed on the epoxy resin cross-linking model. LAMMPS output the bonds.reaxff file at specified trajectory frames. This file records the bonding status of every atom in the current frame. The number of C-O-C and N-C bond breakage events can be calculated by analyzing the bonding information across different frames. Based on the bonds.reaxff file, this study extracted the variation in the number of backbone C-O-C bonds for each output frame. A C-O-C bond breakage event is counted as $N_{breakages\_N-C}$. The calculation formula is given by Equation (1): $N_{original\_C-O-C}$ represents the original number of C-O-C bonds and $N_{current\_C-O-C}$ represents the number of C-O-C bonds in the current frame.

(1) $$N_{breakages\_C-O-C}=N_{current\_C-O-C}-N_{original\_C-O-C}$$

From the above basic cross-linking network structure, it can be known that the amine groups in the curing agent react with two epoxy groups when fully crosslinked. However, under incomplete cross-linking conditions, an NH_2_ group may react with only one epoxy group. Consequently, N atoms can exist bonded to either three carbon atoms (denoted as N-3C) or two carbon atoms (denoted as N-2C). Upon damage to the epoxy resin’s cross-linked structure, both N-3C and N-2C bonds are susceptible to cleavage. Specifically, a single cleavage event in an N-3C bond reduces it to N-2C, while a second cleavage event leads to its complete dissociation. In contrast, a single cleavage event in an N-2C bond results in its complete dissociation. Based on the above analysis, the number of N-C bond breaks in each frame is recorded as $N_{breakages\_N-C}$, and its calculation method is as shown in Equation (2), where $N_{original\_N-3C}$ and $N_{original\_N-2C}$ represent the initial number of N-3C bonds and N-2C bonds. And $N_{current\_N-3C}$, $N_{current\_N-2C}$ respectively represent the number of N-3C bonds and N-2C bonds in this frame.

(2) $$N_{breakages\_N-C}=\left(N_{original\_N-3C}\times 2+N_{original\_N-2C}\right)-\left(N_{current\_N-3C}\times 2+N_{current\_N-2C}\right)$$

The extent of damage to the cross-linking structure of epoxy resin can be quantified by calculating the changes in the number of C-O-C bonds and N-C bonds. Furthermore, the number of intact C-O-C bonds and N-C bonds remaining after the simulation’s conclusion can directly reflect the integrity of the epoxy resin’s cross-linking structure. Consequently, this study proposes the integrity of the epoxy resin cross-linking structure integrity $I_{cross\_integrity}$ as a microscopic damage indicator for epoxy resin. The integrity of the epoxy resin cross-linking structure is quantified by the ratio of the number of remaining C-O-C bonds and N-C bonds at the conclusion of the simulation to the initial number. The calculation method is as Equation (3): $N_{original\_total}$ represents the sum of the initial numbers of the two types of cross-links. $N_{breakages\_end\_C-O-C}$ and $N_{breakages\_end\_N-C}$ respectively represent the number of C-O-C bond and N-C bond breakages at the conclusion of the simulation.

(3) $$\left\{\begin{array}{c} N_{original\_total}=N_{original\_C-O-C}+N_{original\_N-3C}\times 2+N_{original\_N-2C}\\ I_{cross\_integrity}=\frac{N_{original\_total}-(N_{breakages\_end\_N-C}+N_{breakages\_end\_C-O-C})}{N_{original\_total}} \end{array}\right.$$

### 3.2. Calculation of Cross-Linking Structural Integrity of Different GQD-Doped Materials

#### 3.2.1. Under CH_2_ Radical

**Figure 6:**
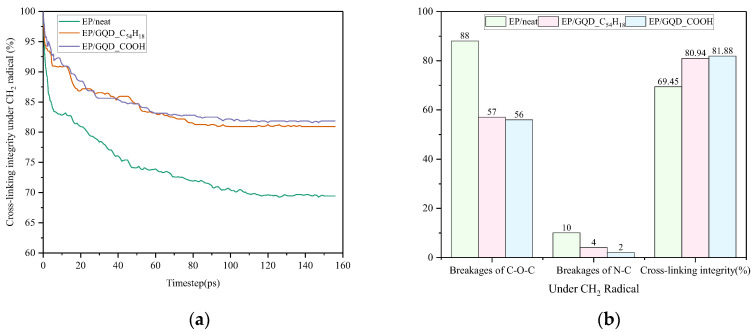
Changes in the cross-linking structure of epoxy resin under CH_2_ radicals. (**a**) The integrity of the cross-linking structure of three materials. (**b**) The final number of C-O-C bond and N-C bond breaks and the integrity of the cross-linking structure of the three materials.

According to the calculation formula in Section 3.1, the changes in the cross-linking structural integrity of the epoxy resin undoped model and the two different doped models under three types of radicals over time were calculated separately. The calculation results for the three materials EP/neat, EP/GQD_C_54_H_18_, and EP/GQD_COOH under CH_2_ radicals are shown in Figure 6. As demonstrated in Figure 6a, under conditions of CH_2_ radical damage, the trends in cross-linking structural integrity for the three materials are nearly identical, exhibiting a rapid decline in the initial 20 ps. The decline rate for the epoxy resin without GQD doping is significantly higher than that of the other two doped models. As time increases to 80 ps, the cross-linking structural integrity of the EP/GQD_C_54_H_18_ and EP/GQD_COOH samples tends to stabilize. In contrast, the cross-linking structural integrity of the EP/neat sample continues to decrease until it reaches a state of stability at 110 ps. The rate of decrease in the integrity of the cross-linking structure of EP/GQD_C_54_H_18_ and EP/GQD_COOH was significantly lower than that of EP/neat, and the final stable value was higher than that of EP/neat. This means that the doping of GQDs can effectively suppress the destruction of the cross-linked structure of epoxy resin by CH_2_ radicals.

Figure 6b presents a statistical analysis of the number of C-O-C bond and N-C bond breaks, as well as the integrity of the cross-linking structure at the conclusion of the simulation. A notable observation is that the variation in the number of N-C bond breaks is significantly smaller than that of C-O-C bonds. The primary reason for this phenomenon is the inherent disparity in the number of N-C bonds and C-O-C bonds within the cross-linking structure of epoxy resin [29,30]. The latter exhibits a greater initial quantity and is more prone to fracture, resulting in more pronounced changes from a statistical, mathematical perspective. However, rupturing C-O-C bonds is equally significant to rupturing N-C bonds for the integrity of the epoxy resin’s cross-linked structure: rupturing either type of bond will damage the cross-linking network. Compared to EP/neat, the number of C-O-C bond and N-C bond breaks under EP/GQD_C_54_H_18_ and EP/GQD_COOH conditions is significantly lower, resulting in a notable improvement in the integrity of the cross-linking structure. The presence of doped materials, designated as EP/GQD_C54H18 and EP/GQD_COOH, lead to a significant decrease in the number of C-O-C bond breaks, with respective reductions of 35.23% and 36.36%. Concurrently, a substantial decline in N-C bond breaks is observed, reaching 60% and 80%. The integrity of the cross-linked structure demonstrated a 11.49% and 12.43% improvement, respectively. From the above data analysis, it can be concluded that under CH_2_ radical conditions, the two different terminally modified GQDs exhibit nearly identical resistance to CH_2_ radical damage, with the GQDs modified with a terminal -COOH group showing slightly higher resistance than the GQDs modified with a terminal H group.

#### 3.2.2. Under CH_2_O Radical

**Figure 7:**
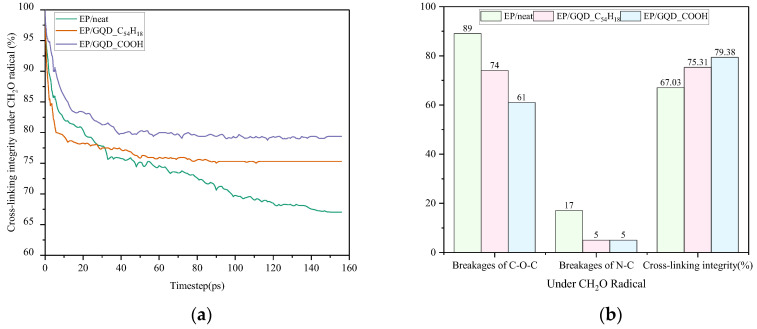
Changes in the cross-linking structure of epoxy resin under CH_2_O radicals. (**a**) The integrity of the cross-linking structure of three materials. (**b**) The final number of C-O-C bond and N-C bond breaks and the integrity of the cross-linking structure of the three materials.

Figure 7 illustrates the alterations in pertinent parameters resulting from CH_2_O radical damage. In contrast to the trend observed under CH_2_ radical damage, the cross-linking structural integrity of EP/GQD_C_54_H_18_ in Figure 7a exhibits a faster rate of decrease than that of EP/neat in the initial stage, but stabilizes in the middle stage, ceased to decline, and ultimately exceeds that of EP/neat. The underlying cause of this phenomenon is attributed to the initial attraction of oxygen-containing free radicals, such as CH_2_O, by GQD_C_54_H_18_, which possesses H at both ends [31]. This results in a local free radical concentration that exceeds the EP/neat value for a brief period. However, when attacked by CH_2_ radicals, the unpaired electrons of the CH_2_ radical are concentrated on the C atom, which may facilitate the formation of an addition reaction with the GQD structure, thereby suppressing radical activity at an early stage. But the unpaired electrons in CH_2_O are present on both the C and O atoms, making them difficult to neutralize directly. Additionally, the C-H bond terminals of GQD_C_54_H_18_ struggle to rapidly donate hydrogen to react with the radical, preventing the formation of a stable structure [32], leading to the highly concentrated CH_2_O radicals instead disrupting the relatively weak C-O-C bond structure in the epoxy resin. As the reaction proceeds, GQD_C_54_H_18_ begins to release H radicals, and active species such as CH_2_O are consumed in the middle stage, thereby inhibiting further damage to the epoxy resin. However, EP/GQD_COOH does not exhibit such phenomena. The initial decrease in cross-linking structural integrity of EP/GQD_COOH is slower than that of EP/neat. This phenomenon can be attributed to the -COOH-modified GQD terminals, which possess a greater number of active sites, thereby facilitating hydrogen donation. The hydrogen donors formed by covalent bond defects of -COOH groups on the GQD_COOH surface can more effectively inhibit free radical activity [33]. As shown in Figure 7b, the cross-linking structural integrity of EP/neat under CH_2_O radical damage is 67.03%. The final cross-linking structural integrity of EP/GQD_C_54_H_18_ and EP/GQD_COOH exhibited final cross-linking structural integrity of 75.31% and 79.38%, respectively, representing increases of 8.28% and 12.35% compared to EP/neat. A comparison of the CH_2_ radicals reveals that EP/GQD_COOH exerts a more pronounced effect against oxygen-containing radicals in comparison to EP/GQD_C_54_H_18_.

#### 3.2.3. Under CH_2_OO Radical

**Figure 8:**
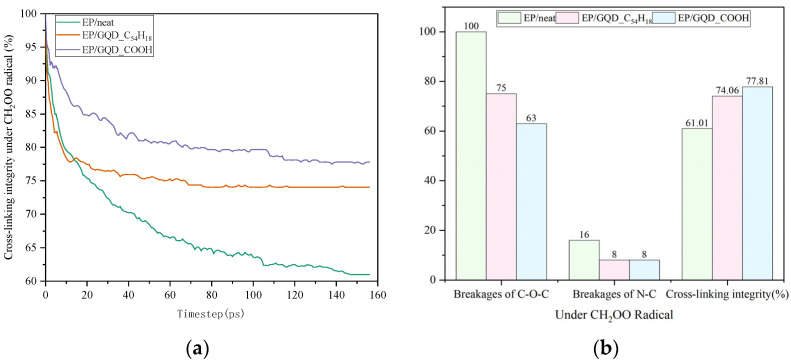
Changes in the cross-linking structure of epoxy resin under CH_2_OO radicals. (**a**) The integrity of the cross-linking structure of three materials. (**b**) The final number of C-O-C bond and N-C bond breaks and the integrity of the cross-linking structure of the three materials.

The damage to epoxy resin under CH_2_OO radicals is shown in Figure 8. The damage trend of epoxy resin under CH_2_OO peroxyl is highly similar to that under CH_2_O. In Figure 8a, EP/GQD_C_54_H_18_ initially exhibits a slightly higher rate of decrease in cross-linking structural integrity due to the difficulty in rapidly neutralizing oxygen-containing free radicals, compared to EP/neat. However, after 20 ps, EP/neat continues to decline rapidly, while EP/GQD_C_54_H_18_ begins to stabilize. EP/GQD_COOH has been demonstrated to effectively limit radical activity from the initial stage, resulting in a significantly lower rate and extent of decrease in cross-linking structural integrity. Epoxy resin without GQDs takes much longer to stabilize when attacked by free radicals than epoxy resin with GQDs, proving that free radicals remain active for a long time inside pure epoxy resin, continuously destroying the epoxy resin cross-linking structure. The presence of GQDs effectively limits the activity of free radicals, thereby achieving the function of resisting free radical damage.

### 3.3. Radiation Resistance Analysis of Different Materials Under Various Free Radicals

As demonstrated in the preceding analysis, various materials demonstrate disparate levels of resistance to free radical damage under distinct free radical conditions, with the ultimate cross-linking structural integrity exhibited in Table 1. The three types of free radicals possess different abilities to damage epoxy resin. Oxygen-containing free radicals have been demonstrated to possess a greater capacity for damage when compared to alkyl radicals, with peroxyl exhibiting the most significant destructive potential. The damage caused by peroxyl resulted in the lowest cross-linking structural integrity of all three materials, with the final integrity of EP/neat retaining only 61.01%.

In the context of CH_2_ radical damage, EP/GQD_C_54_H_18_ and EP/GQD_COOH exhibit comparable levels of protection for the epoxy resin cross-linked structure, exhibiting a discrepancy of merely 0.94%. This observation suggests that, under alkyl action, the presence of functional group modification on the GQDs terminal has a negligible impact on its protective capacity. However, when targeting oxygen free radicals, functional group-modified GQDs show a more obvious advantage. In CH_2_O, the cross-linking structural integrity of EP/GQD_COOH is 4.07% higher than that of EP/GQD_ C_54_H_18_, and this characteristic is 3.75% higher in CH_2_OO. Consequently, when exposed to oxygen free radicals, EP/GQD_COOH with functional group modification exhibited stronger resistance to free radical damage than EP/GQD_C_54_H_18_. Moreover, in the context of peroxyl groups that induce damage, EP/GQD_COOH exhibits superior inhibitory capacity, enhancing cross-link structure integrity by 16.8%. Since epoxy resin may generate various free radicals after irradiation, if the alkyl content in the free radicals is high, both GQD_C_54_H_18_ and GQD_COOH can be used as dopant materials. If the content of oxygen-containing free radicals is high, GQD with functional group modification should be prioritized for doping modification of epoxy resin, as the functional groups at the terminal of GQD have a more significant effect in inhibiting free radicals.

## 4. Conclusions

This study investigated the damage behavior of EP/neat, EP/GQD_C_54_H_18_ (with H at ends), and EP/GQD_COOH (with functional groups) under the attack of three different free radicals (CH_2_, CH_2_O, CH_2_OO) induced by irradiation at high altitudes. It also proposes the concept of cross-linking structural integrity as a microscopic indicator to compare and analyze their resistance to free radical damage before and after GQD doping.

The findings suggest that GQDs, owing to their distinctive sp^2^ hybrid configuration, possess the capacity to impede the degradation of epoxy resin by free radicals and enhance the integrity of the epoxy resin cross-linking structure. When exposed to alkyls, the two GQD-doped materials exhibited significant and similar protective effects, with little influence from the terminal functional groups. The discrepancy in the enhancement of cross-linking structural integrity between the two materials was minimal, with a difference of only 0.94%. However, when faced with more reactive oxygen-containing free radicals (CH_2_O, CH_2_OO), GQDs with functional groups at their ends perform better. GQD_C_54_H_18_, which has hydrogen at its end, initially attracts and locally enriches oxygen-containing free radicals, which slightly exacerbates the initial damage to the material, but then begins to inhibit free radical activity in the middle stage. In contrast, GQD_COOH with carboxyl groups at the ends, leveraging its excellent hydrogen-donating capability, can rapidly suppress radicals, thereby providing more effective protection throughout the process and exhibiting the best damage resistance performance. Its cross-linking structural integrity under oxygen-containing radical damage is approximately 3.75% to 4.01% higher than that of GQD_C_54_H_18_.

In summary, GQDs have been demonstrated to exhibit a significant inhibitory effect on free radicals generated by irradiation in high-altitude regions. When selecting GQD modification strategies for practical applications, targeted designs can be developed based on the types of free radicals that may be generated in the anticipated irradiation environment. In environments dominated by alkyl radicals, both types of GQD are effective dopants. Conversely, in environments with a high concentration of oxygen-containing radicals, GQD modified with functional groups, such as -COOH, should be prioritized to enhance the radiation resistance of epoxy resin composites. The existing industrial techniques for modifying epoxy resins with GQDs have established a solid foundation. Diverse preparation and control methods offer promising prospects for GQDs modification. The GQD-modified epoxy resin approach proposed herein is feasible for practical application trials, providing a robust basis for designing radiation-resistant modifications of epoxy resins.

## Figures and Tables

**Table 1:** Cross-linking structural integrity of different materials under different free radicals.

	EP/Neat	EP/GQD_C_54_H_18_	EP/GQD_COOH
CH_2_	69.45%	80.94%	81.88%
CH_2_O	67.03%	75.31%	79.38%
CH_2_OO	61.01%	74.06%	77.81%

## Data Availability

The original contributions presented in the study are included in the article, further inquiries can be directed to the corresponding author.
